# Macrophages Loaded with Fe Nanoparticles for Enhanced Photothermal Ablation of Tumors

**DOI:** 10.3390/jfb13030094

**Published:** 2022-07-14

**Authors:** Lei Yu, Shuntao Zhu, Kun Qin, Xueyu Fan, Lu An

**Affiliations:** 1 Department of Dermatology, Zhujiang Hospital of Southern Medical University, No. 253 Gongye Avenue, Guangzhou 510282, China; xiaoyu927@smu.edu.cn (L.Y.); qinkun2022@foxmail.com (K.Q.); xueyufan2022@163.com (X.F.); 2 The Key Laboratory of Resource Chemistry of the Ministry of Education, Shanghai Normal University, Shanghai 200234, China; shuntaozhu@163.com; 3 The Shanghai Key Laboratory of Rare Earth Functional Materials, Shanghai Normal University, Shanghai 200234, China; 4 The Shanghai Municipal Education Committee Key Laboratory of Molecular Imaging Probes and Sensors, Shanghai Normal University, Shanghai 200234, China

**Keywords:** iron magnetic nanoparticles, macrophages, nanoparticles delivery, photothermal therapy, MRI

## Abstract

Magnetic iron nanoparticle-based theranostics agents have attracted much attention due to their good magnetism and biocompatibility. However, efficiently enriching tumors with iron nanoparticles to enhance the treatment effect remains a pressing challenge. Herein, based on the targeting and high phagocytosis of macrophages, an Fe nanoparticle-loaded macrophage delivery system was designed and constructed to efficiently deliver iron nanoparticles to tumors. Hydrophilic Fe@Fe_3_O_4_ nanoparticles with a core-shell structure were synthesized by pyrolysis and ligand exchange strategy. Subsequently, they were loaded into macrophages (RAW264.7 cells) using a co-incubation method. After loading into RAW264.7, the photothermal performance of Fe@Fe_3_O_4_ nanoparticles were significantly enhanced. In addition, Fe@Fe_3_O_4_ nanoparticles loaded into the macrophage RAW264.7 (Fe@Fe_3_O_4_@RAW) exhibited a good *T*_2_-weighted MRI contrast effect and clear tumor imaging in vivo due to the tumor targeting tendency of macrophages. More importantly, after being intravenously injected with Fe@Fe_3_O_4_@RAW and subjected to laser irradiation, the tumor growth was effectively inhibited, indicating that macrophage loading could enhance the tumor photothermal ablation ability of Fe@Fe_3_O_4_. The macrophage mediated delivery strategy for Fe@Fe_3_O_4_ nanoparticles was able to enhance the treatment effect, and has great potential in tumor theranostics.

## 1. Introduction

Iron-based magnetic nanoparticles are considered excellent theranostics agents, which are widely used for tumor imaging and hyperthermia therapy due to their good biocompatibility and easy surface modification [1,2,3,4,5]. For example, iron-based nanoparticles are used in T_2_-weighted magnetic resonance imaging (MRI) and magnetic hyperthermia therapy due to their good magnetic properties, while their strong absorption in the near-infrared region also enables their use as good photoacoustic imaging and photothermal therapy agents [6,7,8,9]. A prerequisite for tumor theranostics is the efficient delivery of iron-based nanoparticles to tumors [10,11]. To date, a commonly used delivery strategy for iron-based nanoparticles is passive targeting delivery based on the enhanced permeability and retention (EPR) effect of tumors [12,13]. However, the delivery of iron-based nanoparticles based on the EPR effect is very easily obstructed by the reticuloendothelial system in vivo, resulting in the delivery efficiency of iron-based nanoparticles being very low [14,15]. Therefore, developing a new delivery strategy for iron-based nanoparticles to enhance the treatment effect of tumors is urgently required.

Cell-mediated nanoparticle delivery strategies have great potential in cancer theranostics, since they not only exhibit low biological toxicity but can also penetrate various biological barriers [16,17,18,19,20]. Among all the cells used for nanoparticle delivery, macrophages have attracted increasing attention due to two major advantages [21,22]. One is that macrophages can target cancer-related cytokines and chemokines, allowing them to specifically target and enrich tumors, and the other is that macrophages have a strong phagocytic ability, thus allowing them to load more nanoparticles compared with other cell carriers [23,24,25]. Therefore, macrophages are good candidate vehicles for the delivery of nanoparticles to cancer [26,27,28]. Based on this, Wang et al. reported that macrophages loaded with supramolecular aggregates of CuS nanoparticles can enhance tumor deposition and photothermal therapy [29]. Therefore, the macrophage-mediated delivery strategy will be ideal for the delivery of Fe nanoparticles; however, to the best of our knowledge, few references have been reported.

**Scheme 1:**
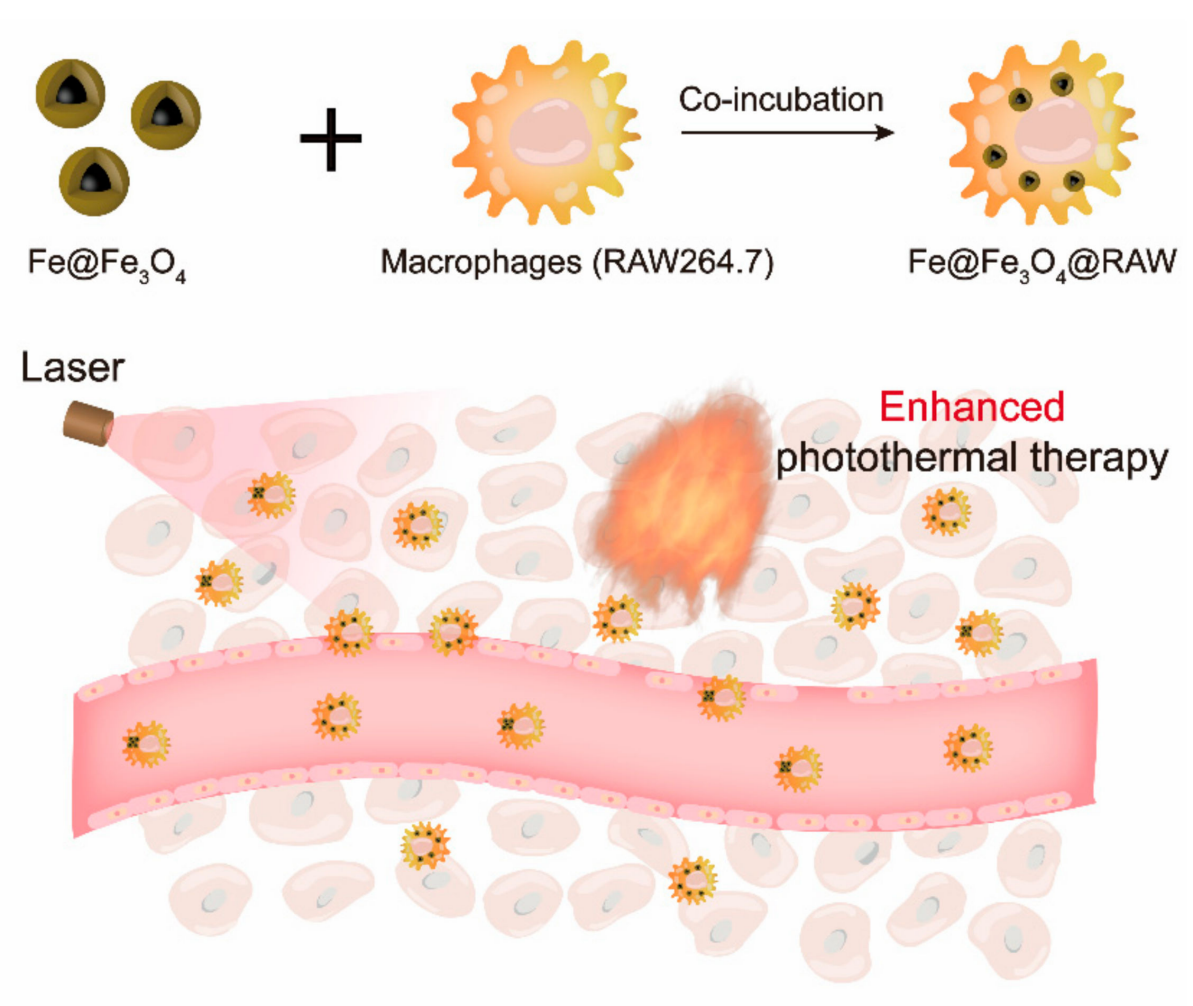
Macrophages loaded with Fe nanoparticles for an enhanced photothermal ablation of tumors.

As a proof of concept, macrophages loaded with Fe-based nanoparticles were designed to demonstrate their enhanced photothermal therapy efficacy (Scheme 1). Firstly, hydrophobic Fe-based nanoparticles were prepared by a pyrolysis method. For bioapplications, the hydrophobic Fe nanoparticles required modification through ligand exchange to obtain hydrophilic Fe nanoparticles, which were subsequently loaded into macrophages (RAW264.7 cells) using a co-incubation method. Lastly, the photothermal and MRI performance of the RAW264.7 cells loaded with Fe nanoparticles were explored both in vitro and in vivo. This delivery strategy takes advantage of the targeting and high phagocytosis of macrophages, which can achieve the integration of tumor diagnosis and treatment, and has the potential to be applied to a variety of therapies.

## 2. Materials and Methods

### 2.1. Materials and Characterization

Fe(CO)_5_ was purchased from Kegonghua Chemical Technology Co., Ltd.(Beijing, China); 1-hexadecylamine, oleylamine and oleic acid were purchased from Sigma Aldrich (Taufkirchen, Germany); 1-octadecene and 3-(3,4-dihydroxyphenyl) propionic acid (DHCA) were obtained from Alfa Aesar (Lancashire, UK). All reagents (AR) were purchased and used directly without purification.

The crystalline phase of Fe@Fe_3_O_4_ was analyzed by X-ray powder diffraction (XRD, D/MAX2000, Rigaku, Tokyo, Japan) and the morphology was characterized by transmission electron microscopy (TEM, JEM-2100, JEOL, Tokyo, Japan) and high-resolution transmission electron microscopy (HR-TEM, JEM-2100, JEOL, Tokyo, Japan). The hydrodynamic size distribution and zeta potential of Fe@Fe_3_O_4_ were determined by Zetasizer (Nano-ZS90, Marlven Panalytical, Malvern, UK). The functional groups existing on the surface of Fe@Fe_3_O_4_ were characterized by Fourier transform infrared spectrum (FTIR, Nicolet-iS10, Thermo Fisher, Waltham, MA, USA). The absorbance of various concentrations of Fe@Fe_3_O_4_ at 808 nm was measured by a UV spectrophotometer (DU 730, Beckman Coulter, Indianapolis, IN, USA). The concentration of Fe was determined by inductively coupled plasma-atomic emission spectrometry (ICP-AES, VISTAMPXICP Varian, Palo Alto, CA, USA). Temperature changes were recorded by a thermal camera (A310, FLIR Systems, Wilsonville, OR, USA), magnetic resonance imaging (MRI) in vitro and the relaxivity were obtained by a nuclear magnetic resonance imaging analyzer (0.5 T, NMI20-Ananlyst, NIUMAG, Shanghai, China), and MRI performed in vivo was characterized by a 1.0 T small animal MRI system (MesoMR, NIUMAG, Shanghai, China).

### 2.2. Synthesis of Fe@Fe_3_O_4_ Nanoparticles

Hexadecylamine hydrochloride was prepared using an ice bath as described by [30]. Generally, hydrochloride was mixed with 1-hexadecylamine for 5 h until a white jelly was formed. After extraction with n-hexane and H_2_O, the product was filtrated and dried in a vacuum. The obtained hexadecylamine hydrochloride was mixed with 1-octadecene and oleylamine for 1 h under dehydrated and deoxygenated conditions at room temperature. This was then heated to 120 °C and continued to react for 40 min. Fe(CO)_5_ was added to the high-temperature pyrolysis for 30 min at 185 °C. Subsequently, oleic acid was added for another 10 min, then the reaction was cooled down to room temperature. Finally, the hydrophobic Fe@Fe_3_O_4_ was obtained after 1 h of air oxidation. The hydrophilic Fe@Fe_3_O_4_ was synthesized by a ligand exchange method. Briefly, DHCA was mixed with hydrophobic Fe@Fe_3_O_4_ in tetrahydrofuran for 2–3 h, then NaOH (0.5 M) was added, followed by washing with acetone and H_2_O and two rounds of centrifugation. The obtained hydrophilic Fe@Fe_3_O_4_ nanoparticles were dispersed in H_2_O for subsequent use.

### 2.3. The Cytotoxicity Analysis

The RAW264.7 cell line was provided by the Cell Bank of Chinese Academy of Sciences (Shanghai, China). The cells in the logarithmic phase (10^6^ cells) were incubated with different concentrations of Fe@Fe_3_O_4_ nanoparticles (0, 10, 20, 50, 100, 200, 400, 600, 800, and 1000 μg/mL) for 24 h, then the supernatant was removed and the cells were washed with PBS. After that, the cell viability was determined according to a typical MTT assay (3-(4,5-dimethylthiazol-2-yl)-2,5-diphenyltetrazolium bromide) by the multi-mode microplate reader (Varioskan Flash, Thermo Fisher Scientific, Waltham, MA, USA).

For confocal imaging, RAW264.7 cells were seeded in a confocal petri dish. After the cells adhered to the dish, Fe@Fe_3_O_4_ nanoparticles were added for co-incubation for 4 h, then the supernatant was removed and the cells were stained with Calcein-AM/PI live/dead double staining kit.

### 2.4. Construction of Fe@Fe_3_O_4_@RAW

The hydrophilic Fe@Fe_3_O_4_ nanoparticles (1 mg/mL) were co-incubated with logarithmic macrophages (RAW264.7 cells, 10^6^ cells) for 3 h, followed by the removal of the supernatant, and washing twice with PBS. After digestion by trypsin for 2 min, the suspension was centrifuged to collect Fe@Fe_3_O_4_@RAW. The Fe concentration was determined by ICP-AES.

### 2.5. MRI Analysis

The in vitro MRI, the longitudinal relaxation time (*T*_1_) and the transverse relaxation time (*T*_2_) of Fe@Fe_3_O_4_@RAW nanoparticles with different concentrations (0, 12.5, 25, 50, 100 and 200 μg/mL) were detected in a 0.5 T MRI analyzer. The parameters of *T*_2_-weighted MRI were: TR = 2500 ms, TE = 36 ms, 82 × 120 mm, 220 Hz/Px. The longitudinal relaxivity (*r*_1_) and the transverse relaxivity (*r*_2_) were defined as the slope of the fitting line, with Fe concentration as the abscissa and 1/*T* as the ordinate.

For in vivo MRI, the BALB/c mice were purchased from Shanghai SLAC Laboratory Animal Co., Ltd. (Shanghai, China). After intravenously injecting mice tumors with Fe@Fe_3_O_4_@RAW nanoparticles, MRI was performed in a 1.0 T MRI scanner at different times (0, 3, 5, and 7 h). The parameters of *T*_2_-weighted MRI were: TR = 1500 ms, TE = 50 ms.

### 2.6. Characterization of Photothermal Properties

The Fe@Fe_3_O_4_ suspension with different concentrations (0, 50, 100, 150 and 200 μg/mL) was irradiated with an 808 nm laser (1.0 W/cm^2^) for 900 s, and the temperature change was monitored by a photothermal imager. The photothermal conversion efficiency (*η*) was calculated using:

(1) $$\eta =\frac{hS\;\left(T_{max}-T_{surr}\right)-Q_{dis}}{I\;\left(1-10^{-A_{808}}\right)}$$

where *h* is the heat conductivity, *S* is the surface area, *T_max_* is the equilibrium temperature, *T_surr_* is the initial temperature, *Q_dis_* is the energy converted by water absorbing the light, *I* is the laser power of 808 nm, and *A*_808_ is the absorbance of Fe@Fe_3_O_4_ at 808 nm.

The tumor growth was monitored for 16 days in 4T1 tumor-bearing mice. The mice were randomly divided into three groups (control, Fe@Fe_3_O_4_ and Fe@Fe_3_O_4_@RAW), and were intravenously injected with PBS, Fe@Fe_3_O_4_, and Fe@Fe_3_O_4_@RAW, respectively. At 5 h after injection, the tumors in the right hind flank of the mice were subjected to laser irradiation (the laser irradiation distance was 1 cm, and the light spot area was 1.13 cm^2^), and then one mouse in each group was sacrificed and the tumor was collected for histopathological staining slices. The tumor growths of the other mice were monitored to investigate the photothermal therapy (PTT) effect.

### 2.7. Ethical Statement for the Tumor Model

The construction and use of the 4T1 tumor bearing model was approved by the Animal Ethics Committee of Shanghai Normal University and the guidelines of the Animal Protection and Animal Use Committee were strictly adhered to (22007066, January 2020).

### 2.8. Statistical Analyses

Data were analyzed with one-way ANOVA statistical analyses (multiple comparisons). Statistical significance was set at *p* < 0.05 (* *p* < 0.05, ** *p* < 0.01, *** *p* < 0.001).

## 3. Results and Discussion

### 3.1. Synthesis and Characterization of Fe@Fe_3_O_4_ Nanoparticles

**Figure 1:**
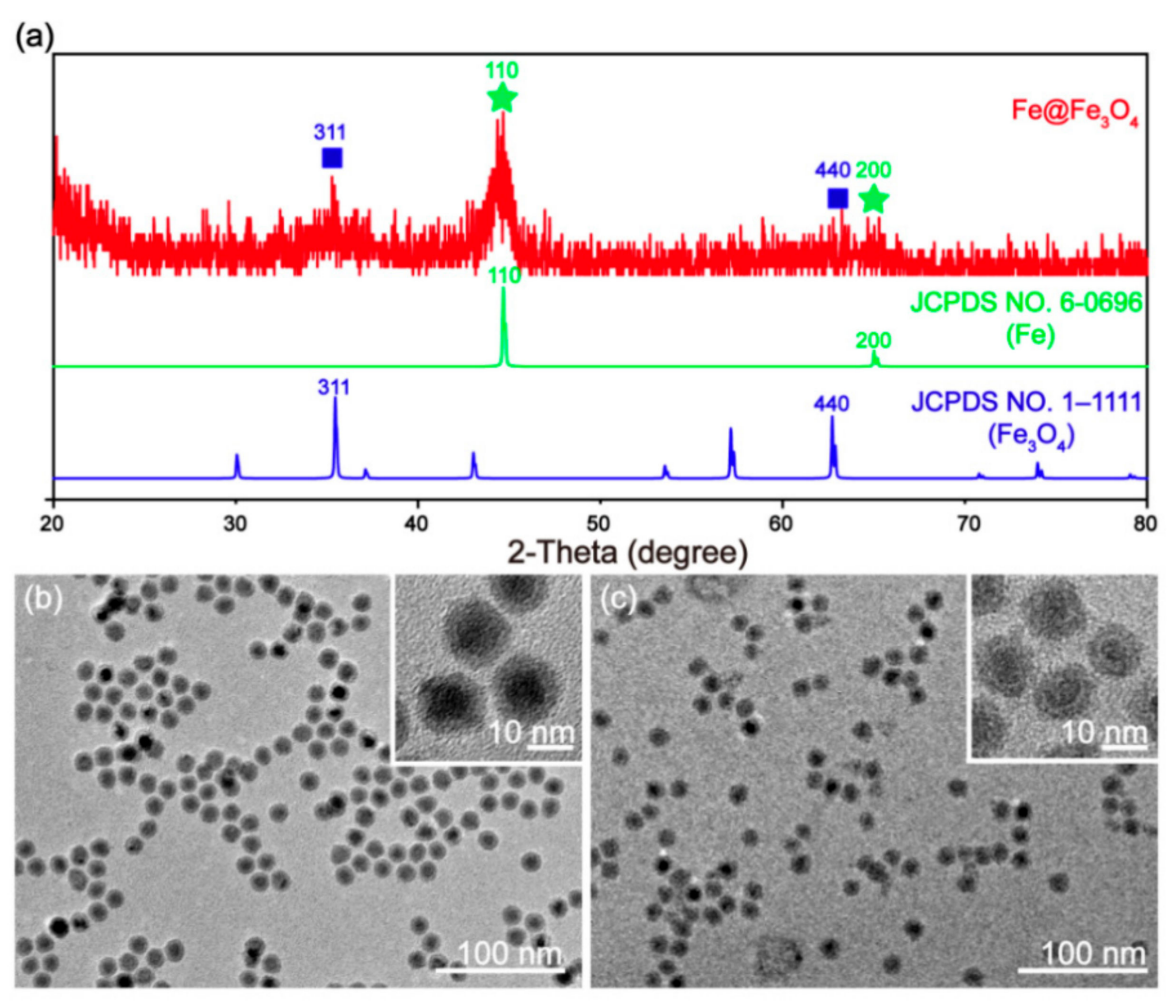
Characterization of Fe@Fe_3_O_4_ nanoparticles. (**a**) XRD pattern of the obtained Fe@Fe_3_O_4_ nanoparticles (red line) and the standard pattern of cubic Fe (green line, JCPDS no. 6-0696) and Fe_3_O_4_ (blue line, JCPDS no. 1-1111). The stars and the squares indicate the characteristic peaks of Fe and Fe_3_O_4_, respectively. TEM and HR-TEM (inset) of the (**b**) hydrophobic and (**c**) hydrophilic Fe@Fe_3_O_4_ nanoparticles.

The hydrophobic Fe-based nanoparticles (Fe@Fe_3_O_4_) were prepared following our previous report [31]. In order to improve their biocompatibility, hydrophilic Fe@Fe_3_O_4_ nanoparticles were further synthesized by a ligand exchange strategy. Firstly, the crystal structure of the obtained Fe@Fe_3_O_4_ nanoparticles was characterized by XRD (Figure 1a). The XRD pattern of the obtained Fe@Fe_3_O_4_ exhibited two obvious peaks at 44.6° and 65.1° which matched well with the 110 and 200 crystal face of cubic Fe, respectively (JCPDS no. 6-0696). In addition, there were two weak peaks at 35.3° and 62.7°, which could be assigned to the 311 and 440 crystal face of cubic Fe_3_O_4_, respectively (JCPDS no. 1-1111). These results demonstrate that the obtained nanoparticles contained both Fe and Fe_3_O_4_ crystals, and that the crystallinity of Fe was good, whereas that of Fe_3_O_4_ was relatively poor. Further, the morphology of the obtained hydrophobic and hydrophilic Fe@Fe_3_O_4_ nanoparticles was observed by TEM. As shown in Figure 1b, the obtained nanoparticles exhibited a core-shell structure with a core diameter of ~10 nm and a shell thickness of ~2 nm (Figure S1a), which can be more clearly observed in the HR-TEM image (Figure 1b inset). The inner core comprised the Fe nanoparticles, and the shell was the Fe_3_O_4_ layer that prevented further oxidation of the Fe core, which was similar to previous results [32]. After ligand exchange (Figure 1c), the morphology and size (Figure S1b) did not change significantly, and the core-shell structure could still be maintained (Figure 1c inset). Due to the presence of DHCA on the surface of Fe@Fe_3_O_4_, the hydrodynamic size was slightly higher than TEM, and the zeta potential was negative, which was consistent with previous results (Figure S2) [31]. Moreover, the two strong vibration peaks at 1575 cm^−1^ and 1404 cm^−1^ originated from the benzene ring of DHCA ligand, and the typical Fe-O stretching vibration peak appeared at 593 cm^−1^ (Figure S3), indicating that the DHCA ligands were modified onto the Fe@Fe_3_O_4_. The above results demonstrate that the hydrophilic Fe@Fe_3_O_4_ nanoparticles were prepared successfully.

### 3.2. Photothermal Performance of Fe@Fe_3_O_4_ Nanoparticles

**Figure 2:**
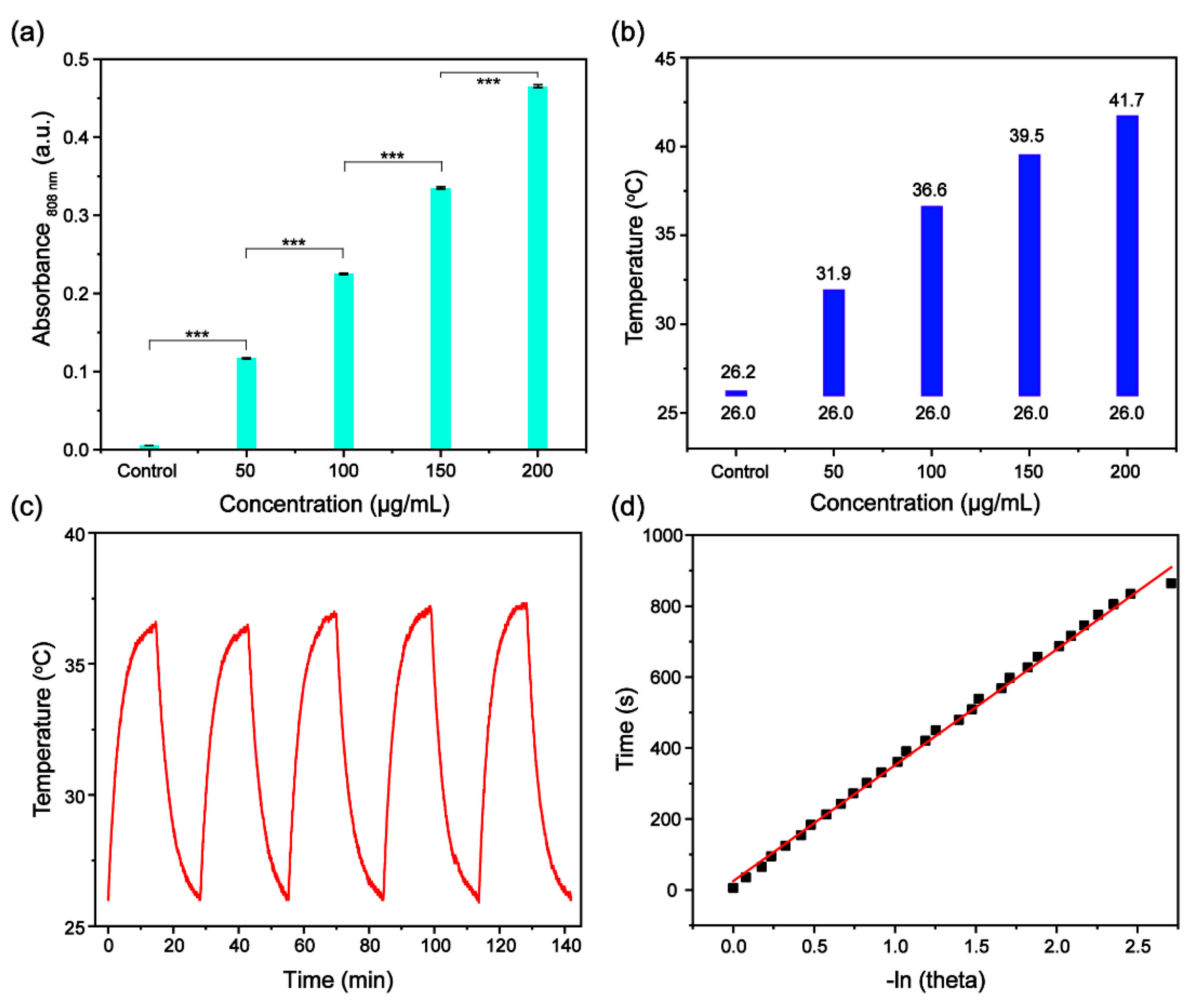
Photothermal performances of the Fe@Fe_3_O_4_ nanoparticles. (**a**) Absorbance at 808 nm of water (control) and Fe@Fe_3_O_4_ nanoparticles water dispersions of different concentrations. *** *p* < 0.001. (**b**) Increasing temperature of the water (control) and Fe@Fe_3_O_4_ nanoparticle water dispersions of different concentrations under 808 nm laser irradiation. (**c**) Photothermal stability of the Fe@Fe_3_O_4_ nanoparticle water dispersion under five cycles of laser on and off. (**d**) Time constant by applying the linear time data versus the negative natural logarithm of the driving force temperature (black square), which was obtained from (**c**).

Considering that the photothermal performance of Fe@Fe_3_O_4_ nanoparticles is related with their absorbance, the absorbance of various concentrations of Fe@Fe_3_O_4_ was first explored (Figure S4a). As shown in Figure 2a, with the increasing concentration (50, 100, 150 and 200 μg/mL), the absorbance of Fe@Fe_3_O_4_ nanoparticles at 808 nm gradually increased. In addition, compared with the control group (water), the temperature of the Fe@Fe_3_O_4_ water dispersion increased dramatically after 808 nm irradiation (1.0 W/cm^2^), and when the concentration reached 100 μg/mL, the temperatures rose by more than 10 °C (Figure 2b and Figure S4b), which was high enough for photothermal therapy (PTT). Moreover, in order to verify the photothermal stability of Fe@Fe_3_O_4_ nanoparticles, the dispersion was irradiated for 15 min, then the irradiation was stopped to allow the sample to cool down to the initial temperature, followed by five cycles of re-irradiation. The heating effect and the maximum reachable temperature (Figure 2c) were essentially the same, indicating the good photothermal stability of the Fe@Fe_3_O_4_ nanoparticles. The absorbance of the Fe@Fe_3_O_4_ nanoparticles changed only slightly before and after laser irradiation (Figure S5), further demonstrating the good photothermal stability. The calculated photothermal conversion efficiency was 34.3% (Figure 2d), which is higher than typical Au nanorods (~22%) [33,34]. All of the above results demonstrate the good photothermal effect of Fe@Fe_3_O_4_ nanoparticles.

### 3.3. Macrophages Loaded with Fe Nanoparticles

**Figure 3:**
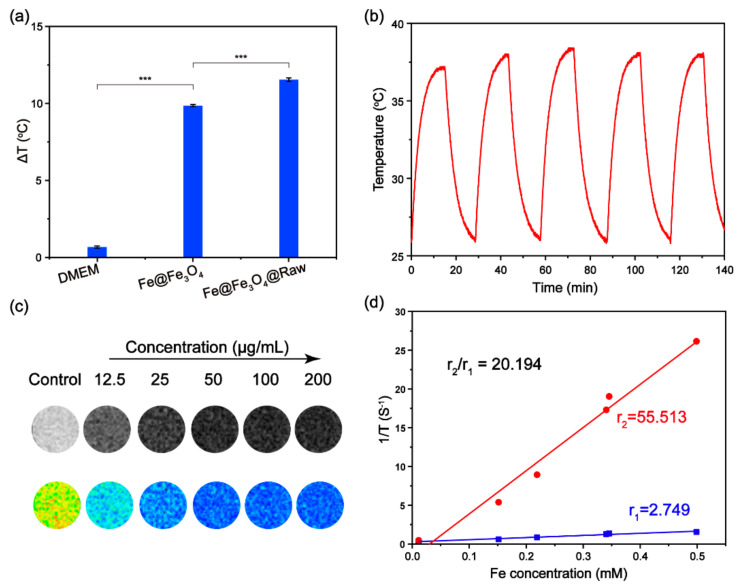
(**a**) Temperature changes (ΔT) of DMEM, Fe@Fe_3_O_4_ nanoparticles (Fe@Fe_3_O_4_) and macrophages loaded with Fe@Fe_3_O_4_ nanoparticles (Fe@Fe_3_O_4_@Raw) under 808 nm laser irradiation. *** *p* < 0.001. (**b**) Photothermal stability of the macrophages loaded with the Fe@Fe_3_O_4_ nanoparticle water dispersion under the five cycles of laser on and off. (**c**) *T*_2_-weighted MR images of water (control) and macrophages loaded with Fe@Fe_3_O_4_ nanoparticles of different concentrations. (**d**) Transverse (*r*_2_, red line) and longitudinal (*r*_1_, blue line) relaxivity of the macrophages loaded with Fe@Fe_3_O_4_ nanoparticles.

To explore the imaging and treatment effect of the Fe@Fe_3_O_4_ nanoparticles delivered by macrophages, the MRI and photothermal performance in vitro should be explored. Before further investigation, the cytotoxicity of Fe@Fe_3_O_4_ nanoparticles to RAW264.7 cells must first be confirmed. As an iron-based material, Fe@Fe_3_O_4_ showed little toxicity, even at very high doses (Figure S6). Meanwhile, obvious green fluorescence (living cells) and weak red fluorescence (dead cells) were observed in the confocal images (Figure S7), indicating that Fe@Fe_3_O_4_ nanoparticles had good biocompatibility. Based on this, RAW 264.7 phagocytic cells loaded with Fe@Fe_3_O_4_ were constructed after 3 h of co-incubation (~717.1 pg/cell), and further irradiated with an 808 nm laser to investigate their photothermal performance (Figure S8). As expected, the macrophages loaded with Fe@Fe_3_O_4_ (Fe@Fe_3_O_4_@RAW) exhibited a greater increase in temperature than the Fe@Fe_3_O_4_ nanoparticles (Figure 3a). In contrast, the temperature of the control group (DMEM) containing cell medium only, barely increased, which ruled out the possibility of DMEM as a solvent interfering with the results. The greater heating effect may have been due to the aggregation of Fe@Fe_3_O_4_ in the macrophages, resulting in locally concentrated heating [35]. Compared with previous reports, the macrophage loading strategy exhibited superior photothermal effect at lower laser power, while macrophages are shown to be excellent carriers for transporting Fe@Fe_3_O_4_ to tumors [36,37]. Moreover, as for Fe@Fe_3_O_4_, after five cycles of laser on and off, the temperature rises of the Fe@Fe_3_O_4_@RAW dispersion remained stable (Figure 3b). All these results prove that Fe@Fe_3_O_4_@RAW is a potential PTT agent with good photothermal stability. Moreover, iron-based nanomaterials have been reported as magnetic resonance imaging (MRI) contrast agents, thus *T*_2_-weighted MRI of Fe@Fe_3_O_4_@RAW was investigated at 0.5 T field strength. As shown in Figure 3c, after being phagocytosed by RAW264.7 cells, Fe@Fe_3_O_4_@RAW with a lower concentration (0~50 μg/mL) exhibited *T*_2_-enhanced contrast enhancement when the concentration was increased. When the concentration was greater than 50 μg/mL, the MR image was no longer darkened, which may have been due to phagocytic saturation of Fe@Fe_3_O_4_ by RAW 264.7 cells. The transverse relaxivity (*r*_2_) and longitudinal relaxivity (*r*_1_) of Fe@Fe_3_O_4_@RAW were 55.513 mM^−1^ s^−1^ and 2.749 mM^−1^ s^−1^, respectively (Figure 3d), which also indicated that it was a good *T*_2_ contrast agent. In addition, *r*_2_ of different patches of Fe@Fe_3_O_4_@RAW with the same Fe concentration was similar (Figure S9), indicating the good reproducibility of RAW loading Fe@Fe_3_O_4_. The above results demonstrate that macrophage phagocytosis could enhance the local aggregation of Fe@Fe_3_O_4_, thus enhancing the photothermal effect. Moreover, because of its good *T*_2_ MRI effect, Fe@Fe_3_O_4_@RAW can be used for MRI-mediated photothermal therapy.

### 3.4. In Vivo Tumor MRI

**Figure 4:**
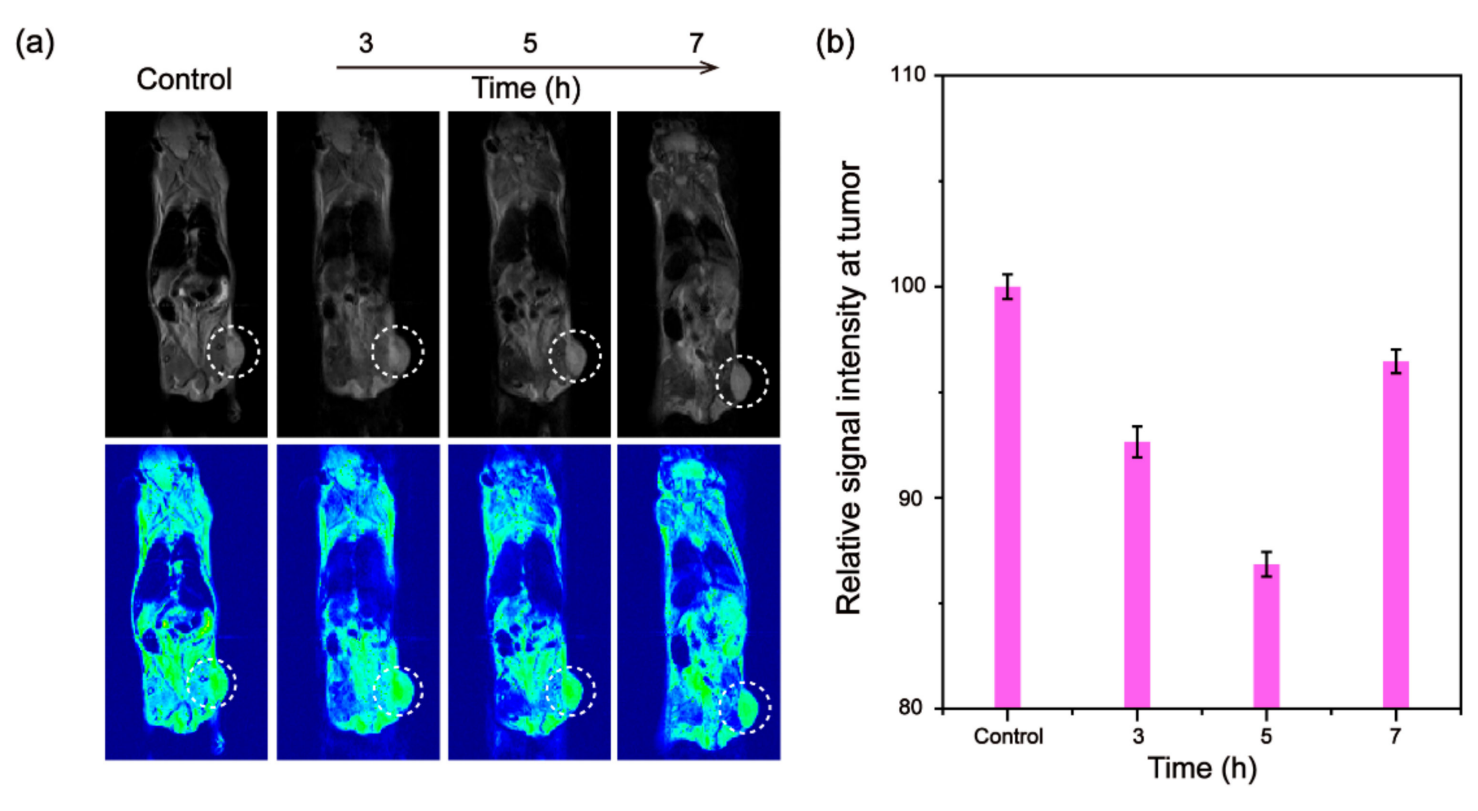
In vivo MRI performance. (**a**) *T*_2_-weighted MR images, and (**b**) corresponding relative signal intensity of tumor, before (control) and after intravenous injection of macrophages loaded with Fe@Fe_3_O_4_ nanoparticles at different time points.

Encouraged by the good *T*_2_ contrast effect of Fe@Fe_3_O_4_@RAW in vitro, the in vivo MRI performance was further explored in 4T1 tumor-bearing mice. As shown in Figure 4a, the tumor site (white circle) was darkened after intravenous injection of Fe@Fe_3_O_4_@RAW and continued to darken 5 h later, indicating the good *T*_2_-weighted MRI contrast effect in vivo. The brightness gradually restored 7 h following injection, which may have been related to the metabolic character of Fe@Fe_3_O_4_@RAW. This suggested that Fe@Fe_3_O_4_@RAW can target the tumor and can be metabolized out of the body. The relative signal intensity at the tumor site can better reflect the changing trend of tumor imaging quantitatively (Figure 4b). The SNR% after intravenous injection of Fe@Fe_3_O_4_@RAW gradually decreased until 5 h post-injection, then increased at 7 h post-injection, which further demonstrates that Fe@Fe_3_O_4_@RAW is a good targeted *T*_2_-weighted MRI contrast agent, and a benefit for tumor theranostics.

### 3.5. In Vivo Photothermal Tumor Ablation

**Figure 5:**
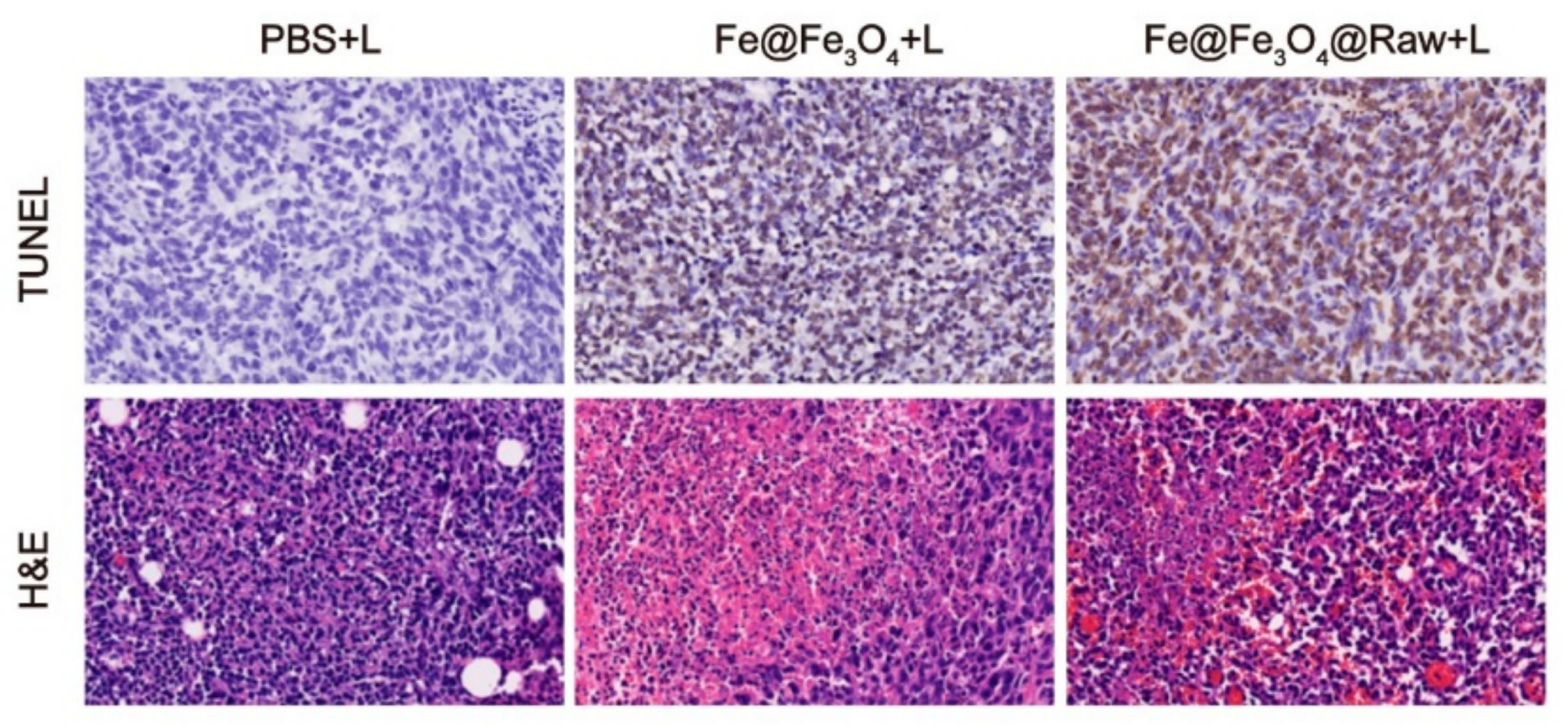
Tumor slice stained by TUNEL and H&E after photothermal therapy.

As mentioned above, macrophage loading can enhance the photothermal effect of Fe@Fe_3_O_4_ in vitro and exhibit tumor-targeting imaging effects, therefore, Fe@Fe_3_O_4_@RAW should be used as a tumor-targeting PTT agent. To prove this speculation, 4T1 tumor-bearing mice were randomly divided into three groups, including control, Fe@Fe_3_O_4_ and Fe@Fe_3_O_4_@RAW groups, each of which was intravenously injected with PBS, Fe@Fe_3_O_4_ and Fe@Fe_3_O_4_@RAW, respectively. Five hours after injection (the best accumulation time at the tumor site), all groups were irradiated with an 808 nm laser for 10 min. Subsequently, one mouse from each group was sacrificed and the tumor tissues were collected and stained with TdT-mediated Dutp Nick-End Labeling (TUNEL) and hematoxylin-eosin (H&E) for histopathological analysis. As shown in Figure 5, the apoptotic cells (brown) appeared in both the Fe@Fe_3_O_4_ and Fe@Fe_3_O_4_@RAW groups, among which the apoptotic were more obvious (78.3%) in the Fe@Fe_3_O_4_@RAW group, demonstrating the more effective tumor PTT effect of Fe@Fe_3_O_4_@RAW. In contrast, after injection with PBS and laser irradiation, most of the cells were normal, indicating the safety of the laser. Similarly, in H&E tumor slices, the morphology of the cells in the Fe@Fe_3_O_4_ and Fe@Fe_3_O_4_@RAW groups was destroyed, and the nucleus was absent, which further proves that the photothermal effect of Fe@Fe_3_O_4_ is better after loading into macrophages.

**Figure 6:**
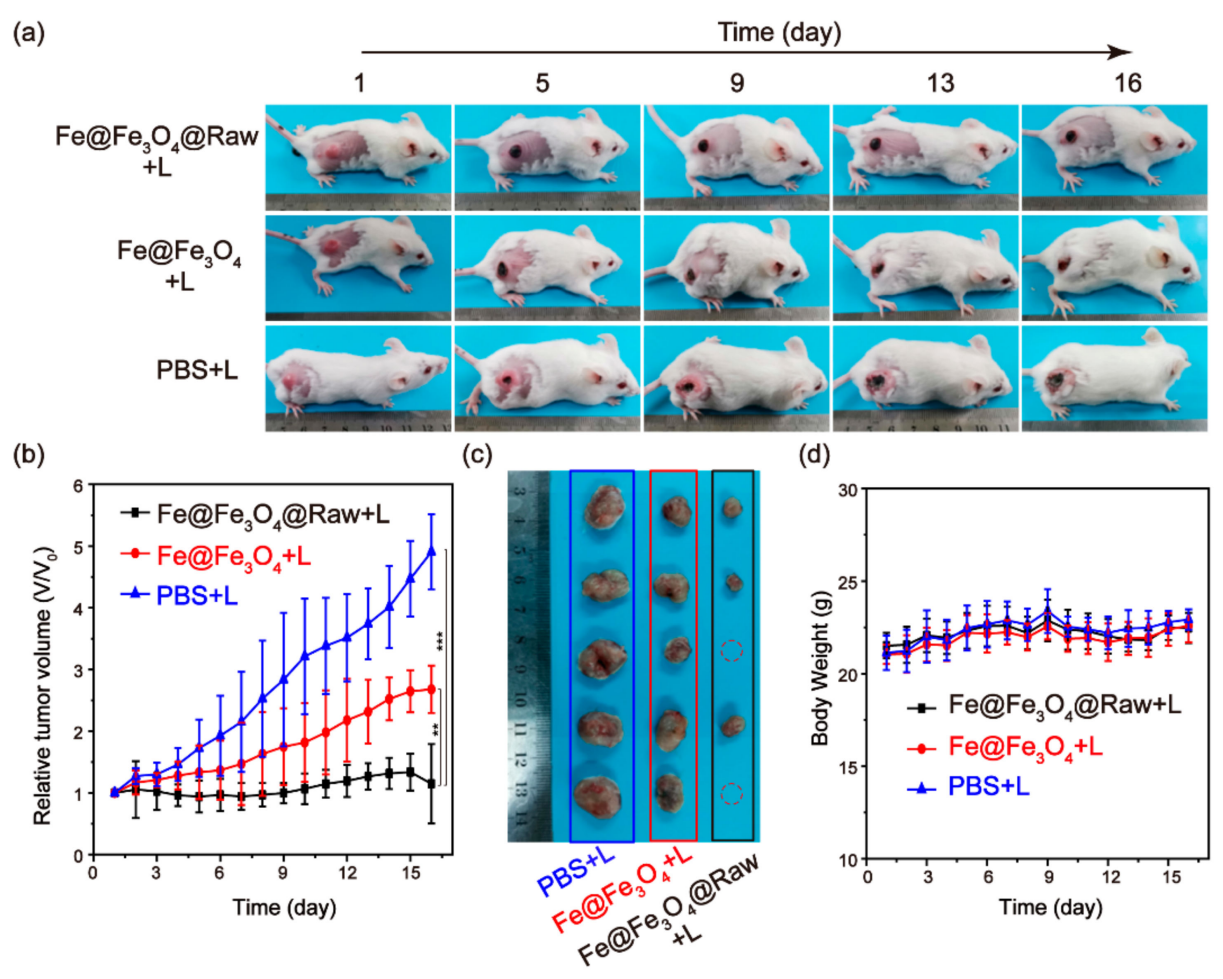
Photothermal therapy effects of the PBS, Fe@Fe_3_O_4_, and Fe@Fe_3_O_4_@RAW groups (n = 5). (**a**) Photographs of the mice in different groups on different days after photothermal therapy. (**b**) Relative tumor volume of the mice in the different groups after photothermal therapy (** *p* < 0.01, *** *p* < 0.001). (**c**) Photographs of the dissected tumors 16 days after photothermal therapy. (**d**) Body weight of the mice in different groups after photothermal therapy.

In addition, the remaining mice from each group were further monitored for 16 days after laser irradiation to evaluate the tumor therapeutic effect. As shown in Figure 6a, after laser irradiation, the tumor sites of the mice in the Fe@Fe_3_O_4_ and Fe@Fe_3_O_4_@RAW groups were blackened, and the tumors were also ablated. However, the tumors in the Fe@Fe_3_O_4_ group began to recur over time, while in the Fe@Fe_3_O_4_@RAW group, tumor growth was completely inhibited and the tumor site was scabbed. In comparison, the tumor growth in the PBS group was still fast, suggesting that the laser alone could not kill tumors. Meanwhile, the change of the relative tumor volume in the Fe@Fe_3_O_4_@RAW group was almost negligible, while that of the Fe@Fe_3_O_4_ group showed a trend of slow growth, and that of the PBS group continued to grow (Figure 6b). Compared with the control group, the dissected tumors in the Fe@Fe_3_O_4_ group were smaller after treatment. It is clear that part of the collected tumors from the Fe@Fe_3_O_4_@RAW group disappeared, meaning that the tumor was ablated and, although they existed, the tumors of the remaining mice were far smaller than those of the other two groups (Figure 6c). All these results further prove the good tumor inhibition effect of Fe@Fe_3_O_4_@RAW. Moreover, the body weight of mice in each group (Figure 6d) remained within a stable range during the treatment period, and no abnormal behavior was observed, which proved that Fe@Fe_3_O_4_@RAW and the laser were safe. This evidence indicates that Fe@Fe_3_O_4_@RAW is a potential PTT agent with good photothermal properties and low biological toxicity.

## 4. Conclusions

In summary, macrophages loaded with Fe@Fe_3_O_4_ nanoparticles were constructed to enhance the photothermal ablation of tumors. Hydrophilic Fe@Fe_3_O_4_ nanoparticles with a core-shell structure were synthesized by a pyrolysis and ligand exchange strategy. After loading into RAW264.7 cells, the local aggregation of Fe@Fe_3_O_4_ nanoparticles resulted in an enhancement of the photothermal effect. In addition, Fe@Fe_3_O_4_@RAW exhibited a good *T*_2_-weighted MRI contrast effect and obvious tumor imaging in vivo due to the tumor targeting tendency of macrophages. More importantly, upon intravenous injection with Fe@Fe_3_O_4_@RAW and subjection to laser irradiation, tumor growth was inhibited effectively, indicating that macrophage loading could enhance the tumor photothermal ablation ability of Fe@Fe_3_O_4_. This delivery strategy takes advantage of the targeting and high phagocytosis of macrophages to achieve the integration of tumor diagnosis and treatment, and has the potential to be applied to a variety of therapies.

## Data Availability

The data presented are available on request from the corresponding author.

## Supplementary Materials

The following supporting information can be downloaded at: https://www.mdpi.com/article/10.3390/jfb13030094/s1, Figure S1: Particle size statistics of Fe@Fe_3_O_4_ nanoparticles; Figure S2: DLS and zeta potential of Fe@Fe_3_O_4_-DHCA; Figure S3: FTIR spectra of Fe@Fe_3_O_4_ nanoparticles, DHCA and Fe@Fe_3_O_4_-DHCA; Figure S4: Absorbance and photothermal performance of hydrophilic Fe@Fe_3_O_4_ nanoparticles; Figure S5: Absorbance of the Fe@Fe_3_O_4_ nanoparticle before and after laser irradiation; Figure S6: The cytotoxicity; Figure S7: The confocal images stained with Calcein-AM/PI; Figure S8: Photothermal performance of DMEM, Fe@Fe_3_O_4_ and Fe@Fe_3_O_4_ @RAW under the irradiation of an 808 nm laser; Figure S9: The repeatability *r*_2_ of Fe@Fe_3_O_4_@RAW with same Fe concentration.
