# Supplementary material for: Macrophages Loaded with Fe Nanoparticles for Enhanced Photothermal Ablation of Tumors
Source: J Funct Biomater. 2022 Jul 14;13(3):94. doi: 10.3390/jfb13030094 (PMC9326737; doi:10.3390/jfb13030094)
Supplement: Supplementary file 1 [file jfb-13-00094-s001.zip › jfb-1769225-supplementary.pdf]

Journal of
Functional Biomaterials

Macrophages Loaded with Fe Nanoparticles for Enhanced Photothermal Ablation of Tumors

Lei Yu [1], Shuntao Zhu [2], Kun Qin [1], Xueyu Fan [1] and Lu An [2,*]

1 Department of Dermatology, Zhujiang Hospital of Southern Medical University, No. 253 Gongye Avenue, Guangzhou 510282, China; xiaoyu927@smu.edu.cn (L.Y.); qinkun2022@foxmail.com (K.Q.); xueyufan2022@163.com (X.-Y.F.)
2 The Key Laboratory of Resource Chemistry of the Ministry of Education, The Shanghai Key Laboratory of Rare Earth Functional Materials, and The Shanghai Municipal Education Committee Key Laboratory of Molecular Imaging Probes and Sensors, Shanghai Normal University, Shanghai 200234, China; shuntaozhu@163.com
* Correspondence: anlu1987@shnu.edu.cn

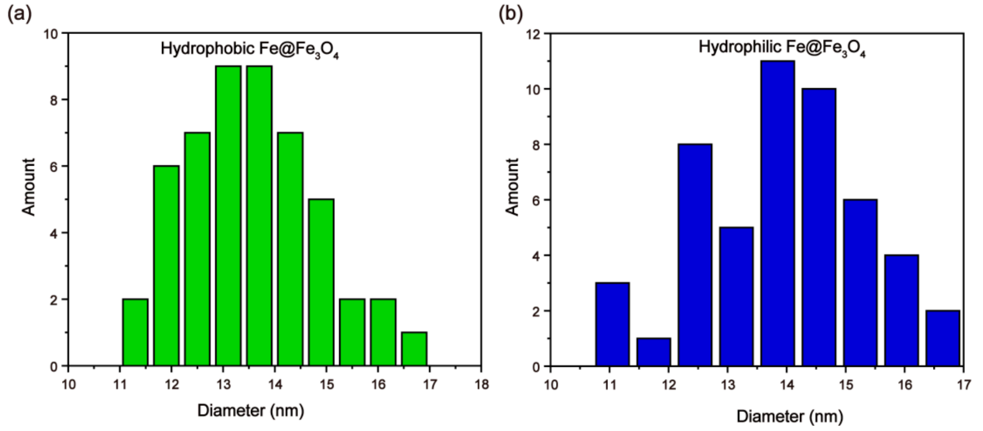

Figure S1. Statistical analysis of particle size for the (**a**) hydrophobic, and (**b**) hydrophilic, $Fe@Fe_3O_4$ nanoparticles based on TEM images from Figure 1b,c.

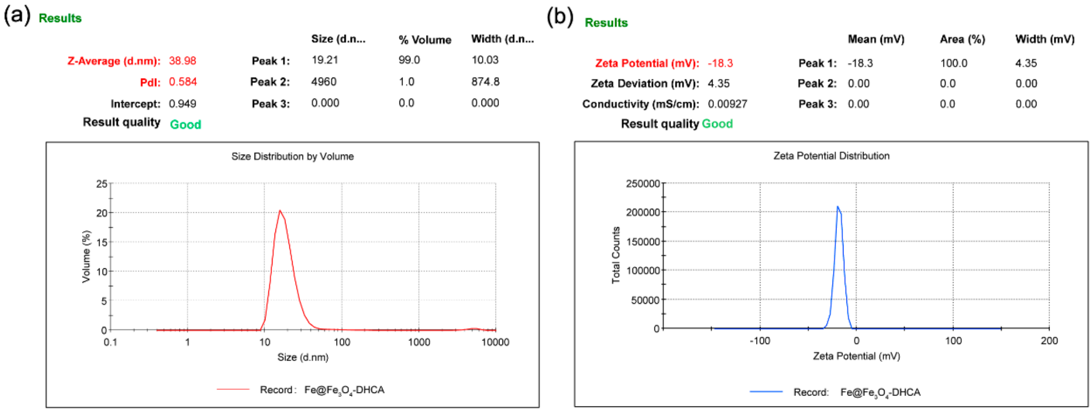

Figure S2. (**a**) The hydrodynamic size distribution, and (**b**) zeta potential, of $Fe@Fe_3O_4$-DHCA nanoparticles.

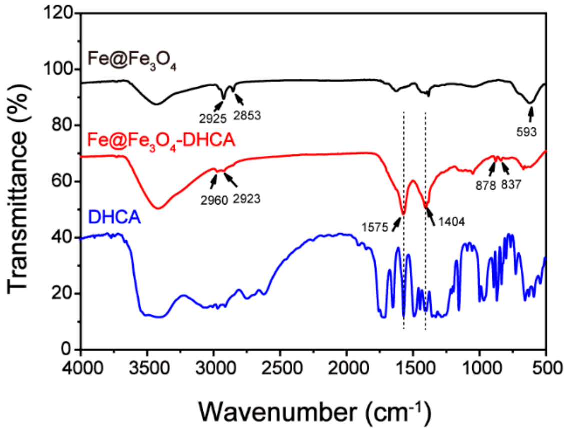

Figure S3. FTIR spectra of hydrophobic Fe@Fe_3O_4 nanoparticles, DHCA and hydrophilic Fe@Fe_3O_4-DHCA.

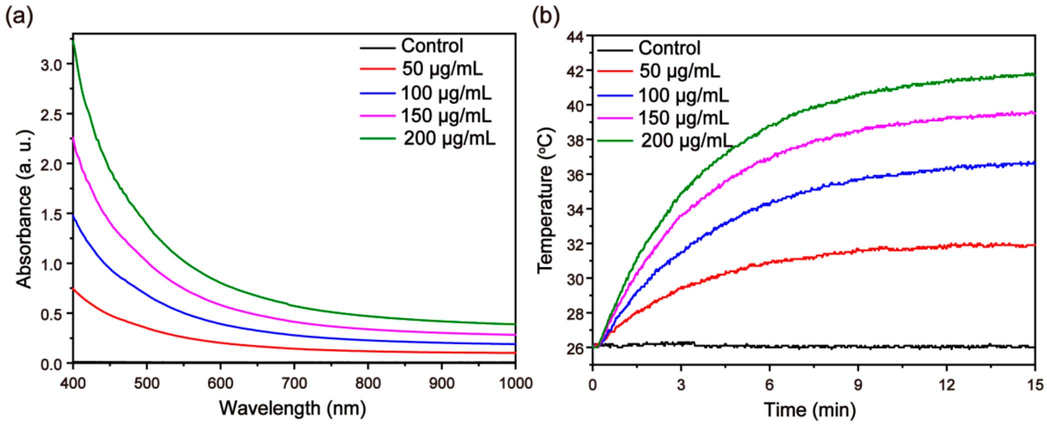

Figure S4. (**a**) Absorbance, and (**b**) photothermal performance, of water (control) and a dispersion of water and hydrophilic Fe@Fe_3O_4 nanoparticles with different concentrations (50, 100, 150 and 200 μg/mL).

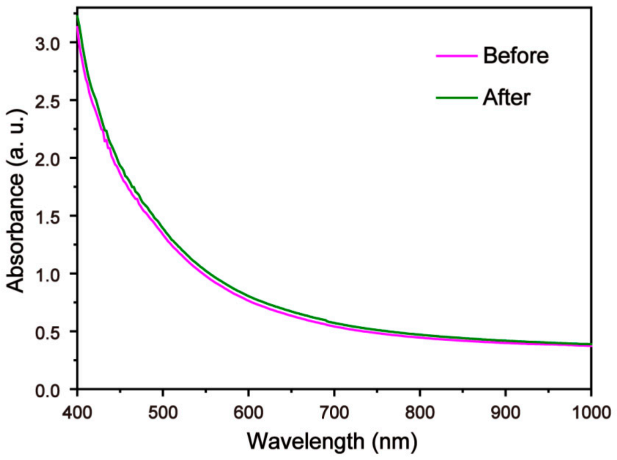

Figure S5. Absorbance of the hydrophilic Fe@Fe_3O_4 nanoparticle water dispersion before (pink line) and after (green line) laser irradiation.

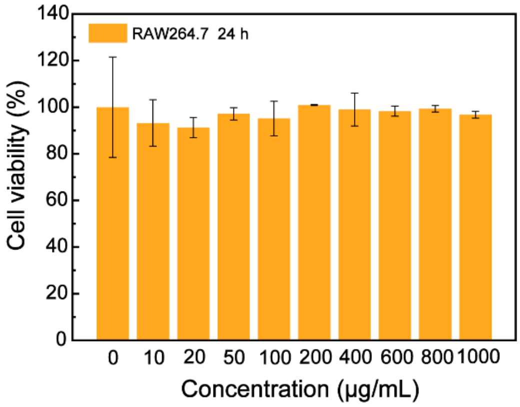

Figure S6. The cytotoxicity of RAW264.7 cells after being incubated with different concentrations of Fe@Fe_3O_4 nanoparticles for 24 h.

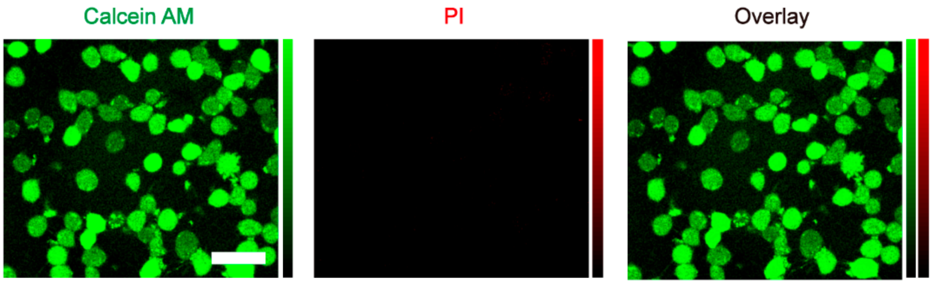

Figure S7. The confocal images of RAW264.7 cells after being incubated with Fe@Fe_3O_4 nanoparticles (1 mg/mL) for 24 h. Scale bar = 25 μm.

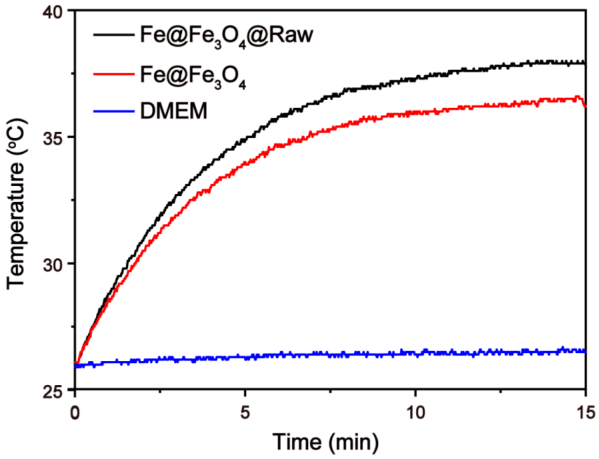

Figure S8. Photothermal performance of DMEM (blue line), Fe@Fe_3O_4 nanoparticles (Fe@Fe_3O_4, red line) and macrophages loaded with Fe@Fe_3O_4 nanoparticles (Fe@Fe_3O_4 @RAW, black line) under the irradiation of an 808 nm laser.

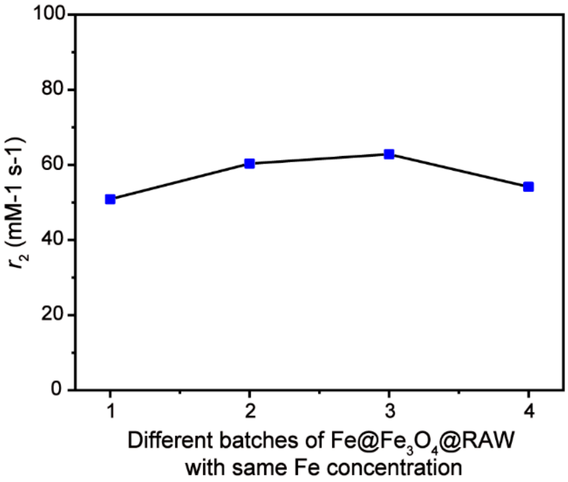

Figure S9. The repeatability transverse relaxivity of Fe@Fe_3O_4@RAW nanoparticles with the same Fe concentration.
